# Identification of Novel Putative Bacterial Feruloyl Esterases From Anaerobic Ecosystems by Use of Whole-Genome Shotgun Metagenomics and Genome Binning

**DOI:** 10.3389/fmicb.2019.02673

**Published:** 2019-11-20

**Authors:** Kamyar Mogodiniyai Kasmaei, John Sundh

**Affiliations:** ^1^Department of Animal Nutrition and Management, Swedish University of Agricultural Sciences, Uppsala, Sweden; ^2^Science for Life Laboratory, Department of Biochemistry and Biophysics, National Bioinformatics Infrastructure Sweden, Stockholm University, Solna, Sweden

**Keywords:** biorefinery, *de novo* assembly, lignocellulosic biomass, phylogenetic analysis, sequence motif, taxonomic classification

## Abstract

Feruloyl esterases (FAEs) can reduce the recalcitrance of lignocellulosic biomass to enzymatic hydrolysis, thereby enhancing biorefinery potentials or animal feeding values of the biomass. In addition, ferulic acid, a product of FAE activity, has applications in pharmaceutical and food/beverage industries. It is therefore of great interest to identify new FAEs to enhance understanding about this enzyme family. For this purpose, we used whole-genome shotgun metagenomics and genome binning to explore rumens of dairy cows, large intestines of horses, sediments of freshwater and forest topsoils to identify novel prokaryotic FAEs and trace the responsible microorganisms. A number of prokaryotic genomes were recovered of which, genomes of *Clostridiales* order and *Candidatus Rhabdochlamydia* genus showed FAE coding capacities. In total, five sequences were deemed as putative FAE. The BLASTP search against non-redundant protein database of NCBI indicated that these putative FAEs represented novel sequences within this enzyme family. The phylogenetic analysis showed that at least three putative sequences shared evolutionary lineage with FAEs of type A and thus could possess specific activities similar to this type of FAEs, something that is not previously found outside fungal kingdom. We nominate *Candidatus Rhabdochlamydia* genus as a novel FAE producing taxonomic unit.

## Introduction

Production of biofuels and biochemicals from lignocellulosic biomass, a non-food renewable carbon resource, has increasingly become of great importance due to increasing global demands for energy and chemicals, increasing prices of fossil fuels and environmental concerns associated with fossil fuels. Lignocellulosic biomass mainly comprises three structural polymers namely cellulose, hemicellulose and lignin. In the cell walls of monocots (e.g., grass, cereals), lignin and hemicellulose interconnect, forming a matrix that encrusts the cellulose (Wong, 2006; Rubin, 2008). This configuration creates a complex structure, believed to be the main cause of recalcitrance of lignocellulosic biomass to enzymatic hydrolysis (Rubin, 2008; Pu et al., 2013).

The linkage between lignin and hemicellulose is mainly mediated by ferulic acid (FA), forming ester bonds with hemicellulose from the carboxylic side and ether bonds with lignin from the phenolic side of the molecule. These ester bonds in the cell walls of plants can be cleaved with feruloyl esterases (FAEs) (EC 3.1.1.73), member of carboxylic ester hydrolases (EC 3.1.1.-) (Jeske et al., 2019), to reduce complexity of cell wall configuration, thereby enhancing utilization of lignocellulosic biomass (Wong, 2006). Further importance of FAEs is in pharmaceutical and food/beverage industries as FA, a product of FAE activity, has evidently antioxidant properties (Pohl and Lin, 2018) and can also be used to produce vanillin (Chen et al., 2016). In addition, several attempts have already been made to improve digestibility of forages in dairy cattle rations by use of FAE producing lactic acid bacteria (Muck et al., 2018).

Feruloyl esterases are classified into four types (A, B, C, and D) based on substrate specificity against model methyl esters and release of diferulic acid (5–5’) from plant cell walls (Crepin et al., 2004). The efficiency of FAEs in breaking lignin-hemicellulose interconnections seems to differ among different FAEs. FAEs-A break these interconnections in the cell walls of cereals at higher rates than FAEs-B (Crepin et al., 2004). Based on phylogenetic analysis, fungal FAEs were classified into seven subfamilies (Benoit et al., 2008) but the phylogeny was further improved in a later attempt, with recognition of 13 subfamilies of fungal FAEs (Dilokpimol et al., 2016). These attempts showed that FAEs did not evolve from a common ancestor (Benoit et al., 2008; Dilokpimol et al., 2016). In a novel approach, protein descriptors, derived from amino acid sequences, were used in conjunction with a machine learning method to classify fungal, bacterial and plant FAEs, which resulted in formation of 12 families of FAEs (Udatha et al., 2011). There is to some extent agreement between the A–D classification and 1–13 subfamily classification as for instance subfamilies 6 and 7 solely include FAEs-B and FAEs-A, respectively. However, the subfamily 1 includes both FAEs-B and FAEs-C and subfamily 5 contains FAEs-A and FAEs-D. It appears that the classification of FAEs can further be improved in the near future when more data is available.

Several fungal and bacterial species are known to produce FAEs, including *Aspergillus* spp., a number of anaerobic fungal species, *Bacillus* spp., *Lactobacillus* spp., etc. (Donaghy et al., 1998; Dilokpimol et al., 2016). Due to industrial significance of FAEs, there is an ever-growing interest to identify new FAEs and new microorganisms with this ability. Potential habitats of FAE producing microorganisms are ecosystems in which, plants are degraded, such as digestive tract of herbivores, soil or aquatic ecosystems. The rapid development of sequencing platforms and metagenomic methodologies has enabled to effectively explore these ecosystems for such purpose. In this work, we explored rumens of dairy cows, large intestines of horses, sediments of freshwater and topsoils of forests by means of whole-genome shotgun metagenomics and genome binning to study prokaryotic capacities for FAE production and potential novelty of the predicted FAEs.

## Materials and Methods

### Sampling

Approximately 50 mL rumen content was sampled from four adult Swedish Red and White breed dairy cows through permanent rumen fistula. Cows had been fed standard diets, containing forage and concentrate, based on their production levels. These cows had been fitted with fistula previously, approved by the Uppsala Ethics Committee (C 93/12 and C 142/14) and were maintained at the Livestock Research Centre of the Swedish University of Agricultural Sciences (SLU) for research/education purposes. Horse fecal samples (ca. 75 g) were directly taken from rectum of four adult horses, fed conventional forage-based diet. These horses were maintained at SLU for research/education purposes approved by the Uppsala Ethics Committee (C 148/13). All the animals used were maintained under SLU policy for use of animals in research and education (SLU.ua 2015.1.1.1-4840). Sediment samples were collected from one stream, one river, one lake and one pond from shallow locations in where, water was still and sediment contained dead plant biomass and thus, sampling locations were considered ecologically similar. Four topsoil samples were obtained from four pine-deciduous forests from locations with decaying plant biomass. All samples were collected in the region of Uppsala, Sweden during spring 2017.

### Library Preparation and Sequencing

DNA extraction was done with NucleoSpin^®^ soil (MACHEREY-NAGEL, Düren, Germany). DNA quality and quantity were checked with Agilent 2200 TapeStation System (Agilent, Santa Clara, CA, United States) and Qubit^®^ 3.0 Fluorometer (Thermo Fisher Scientific, Waltham, MA, United States), respectively by the Science for Life Laboratory (SciLifeLab), Uppsala, Sweden. Library preparation was done with TruSeq DNA PCR-Free kit (Illumina, Inc., San Diego, CA, United States) and paired-end sequencing (2 × 125) was performed using Illumina HiSeq2500 system and v4 sequencing chemistry (Illumina, Inc., San Diego, CA, United States) in one lane by the SciLifeLab.

### Bioinformatic Analysis

#### Assembly and Binning

Reads were quality checked with Trimmomatic (Bolger et al., 2014) (vs. 0.36, LEADING:3, TRAILING:3, SLIDINGWINDOW: 4:15, MINLEN: 36) before pooling into four datasets, referred to as Cow, Horse, Sediment, and Soil. The pooled datasets were *de novo* assembled with Megahit (Li et al., 2015) (vs. v1.1.2, default settings) after which, reads were aligned to contigs ≥1500 bp with bbmap (Bushnell, 2014) (vs. 37.53, default settings). Binning was done with Metabat (Kang et al., 2015) (vs. v2.12.1, minContig: 1500) and bin redundancy was checked by calculating Average Nucleotide Identity (ANI) with FastANI^1^ (vs. v1.1, default setting). A threshold of 95% ANI was used to merge binned genomes. Completeness and contamination of binned genomes were estimated with CheckM (Parks et al., 2015) (vs. v1.0.11) using lineage specific marker genes. Binned genomes with a contamination <10% and a completeness >30% were selected for downstream analyses.

#### Annotation

Annotation of recovered genomes was done with Prokka (Seemann, 2014) (vs. 1.12, default settings) and predicted proteins were further annotated with InterProScan (Jones et al., 2014) (vs. 5.30–69.0, default settings). The InterPro database classifies proteins with similar domains/sites into single entries. The IPR011118 family includes FAE of *faeB* gene (accession ID: AJ309807), tannase and some other proteins. The IPR010126 family contains some lipases, FAE of *faeC* gene (accession ID: AJ505939) and acetyl xylan esterase. The IPR034429 family comprises FAEs-C and IPR002921 domain corresponds to a domain in FAEs-A, similar to a domain in fungal lipases.

Proteins classified as members of IPR011118, IPR034429, IPR010126 or IPR002921 entries were scanned with ScanProsite (de Castro et al., 2006) to identify sequence motifs. They were further queried with BLASTP (vs. 2.7.1, default settings) against a set of reviewed FAEs of the UniProt database (2019-10-03) (The UniProt Consortium, 2019). The protocol used to select these reference FAEs was: searching for “ec:3.1.1.73” at the UniProt database and filtering by “Reviewed.” This resulted in 44 sequences among which, one sequence was incomplete (UniProt ID: P0CT85) and was thus excluded, resulting in total of 41 fungal sequences and 2 bacterial sequences. In addition, the predicted proteins belonging to the FAE-containing entries of InterPro database were subjected to BLASTP search (vs. 2.7.1, default settings) against non-redundant protein database of NCBI (2019-04-03) to assess their novelty.

**FIGURE 1:**
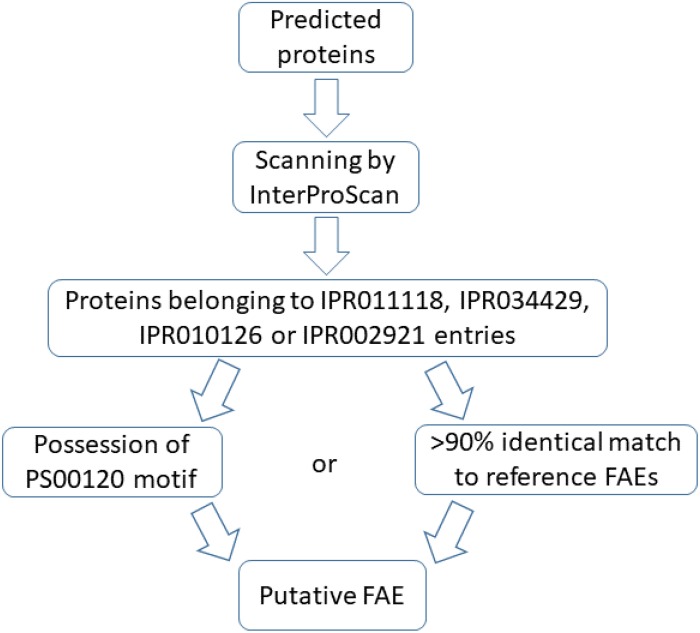
Protocol used to annotate predicted proteins as putative feruloyl esterases (FAEs).

Two conditions were opted for annotation as a putative FAE: more than 90% primary sequence similarity to reference FAEs or possession of the serine active site motif^2^. The consensus pattern of this motif is [LIV]-{KG}-[LIVFY]-[LIVMST]-G-[HYWV]-S-{YAG}-G-[GSTAC], with square and curly brackets indicating acceptable and unacceptable amino acids in the respective positions, respectively. An overview of the annotation protocol is in Figure 1.

#### Community Analysis and Taxonomic Classification

Prokaryotic community composition in pooled datasets was estimated by means of taxonomic classification of reads using Kaiju (Menzel et al., 2016) (vs. 1.7.2, default settings) and non-redundant protein database of NCBI (2019-06-25). Recovered genomes were assigned taxon with phylophlan (Segata et al., 2013) (vs. 0.99, default settings), using predicted proteins from Prokka annotation as input. The taxonomic assignments were further evaluated with CheckM and METAXA2 (Bengtsson-Palme et al., 2015) (vs. 2.2 beta 9, default settings). In case of agreement among predictions, the lowest taxonomic rank given by any of the software was reported and in case of disagreement, the lowest common taxonomic rank was assigned.

#### Phylogenetic Analysis

Signal peptides of putative FAEs were predicted at the SignalP-5.0 server (Almagro Armenteros et al., 2019) and trimmed. The query, reference FAEs (without signal peptides) and three Glucuronoyl esterases (without signal peptides), as an outgroup (Dilokpimol et al., 2016), were aligned with Clustal Omega (Sievers et al., 2011) (vs. 1.2.4) on The European Bioinformatics Institute (EMBL-EBI) Web server (Madeira et al., 2019) using the default settings. Phylogenetic analysis was made with two different methods: maximum likelihood (ML) and neighbor joining (NJ). For the former method, FastTree (Price et al., 2009) (vs. 2.1.10, default settings) was used with 1,000-time resampling and the Shimodaira–Hasegawa test. For the latter, the alignment was first converted to Phylip format on the NGPhylogeny.fr Web server (Lemoine et al., 2019) before using FastME (Lefort et al., 2015) (vs. 2.0) with 1,000-time bootstrapping at the ATGC bioinformatics platform^3^. Both trees were visualized with ETE toolkit (Huerta-Cepas et al., 2016) (vs. 3.1.1).

## Results

### Assembly and Binning

The Cow, Horse, Sediment, and Soil datasets had 2 × 85,285,247, 2 × 46,961,631, 2 × 79,657,128, and 2 × 72,876,571 reads, respectively. Assembly statistics is shown in Table 1. The longest contig was assembled in the Soil dataset with a length of 514,904 bp. More contigs were assembled in the Cow and Horse datasets than in the Sediment and Soil datasets and the Sediment dataset had the poorest assembly statistics. Binning resulted in formation of 87, 83, 15, and 10 binned genomes in the Cow, Horse, Sediment, and Soil datasets, respectively. There was no genome redundancy based on ANI. For downstream analyses, 31, 44, 7, and 6 genomes were selected from the Cow, Horse, Sediment, and Soil datasets, respectively (Supplementary Table S1).

**TABLE 1:** Assembly statistics of Cow, Horse, Sediment, and Soil datasets.

**Co-assembly**	**Number of contigs**	**Max length (bp)**	**N50**	**L50 (bp)**	**N90**	**L90 (bp)**
Cow	157,074	180,624	42,765	2,853	128,498	1,662
Horse	92,420	251,279	19,700	3,548	73,073	1,709
Sediment	25,588	70,080	7,787	2,485	21,338	1,622
Soil	45,259	514,904	13,027	2,546	37,505	1,627

### Community Composition and Recovered Taxa

**FIGURE 2:**
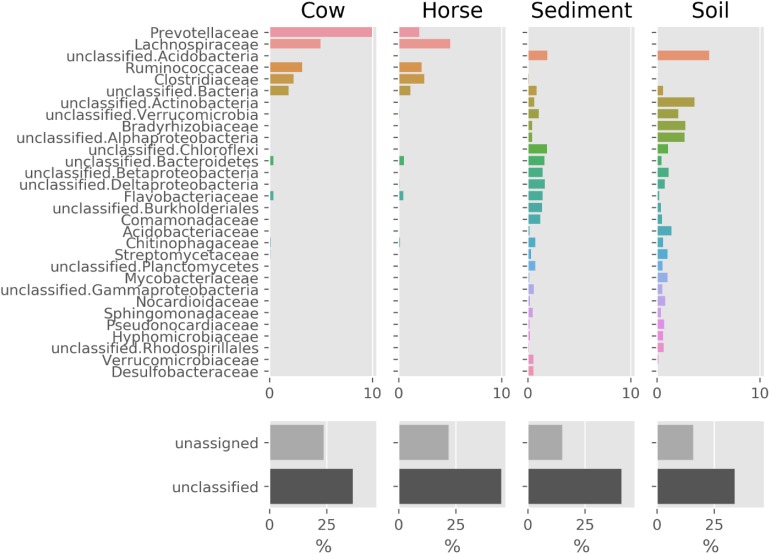
Prokaryotic composition (family level) of Cow, Horse, Sediment, and Soil ecosystems based on classification of reads by Kaiju. Families shown make up at least 75% of the community (together with the unclassified and unassigned sequences). “unclassified” sequences have no classification by Kaiju and “unassigned” sequences are those not assigned to a non-viral species.

The prokaryotic community composition was notably similar between the Cow and Horse samples, with the dominance of *Prevotellaceae*, *Lachnospiraceae*, *Ruminococcaceae*, and *Clostridiaceae* (Figure 2). There were also similarities between the Sediment and Soil samples, with the community comprising a wide range of taxa from *Acidobacteria* and *Actinobacteria* to *Alphaproteobacteria* and *Betaproteobacteria* (Figure 2). Viruses and archaea accounted for a combined total of at most 1.2% in each sample (data not shown).

**FIGURE 3:**
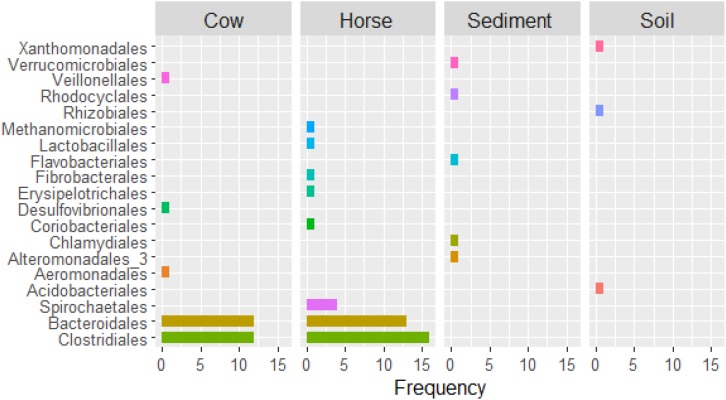
Number of binned genomes per taxon (order level) recovered from Cow, Horse, Sediment, and Soil datasets.

In the Cow and Horse datasets, high (≥90% completeness) and/or low (<90% completeness) quality genomes of *Lachnospiraceae* family (e.g., Cow.1, Cow.7, Horse.8, and Horse.31), within the *Clostridiales* order, and *Prevotellaceae* family (e.g., Cow.2, Cow.19, Horse.5, and Horse.40), within the *Bacteroidales* order, were frequently recovered (Supplementary Table S1). High and/or low quality genomes of *Ruminococcaceae* family, within the *Clostridiales* order, were also frequent in the Horse dataset (Horse.7, Horse.14, and Horse.24). In the Sediment and Soil datasets, high and/or low quality genomes of bacteria typically inhabiting fresh water (e.g., Sediment.1) and soil (e.g., Soil.1 and Soil.4) were reconstructed. Archaeal genomes of *Euryarchaeota* phylum (Cow.14) and *Methanomicrobiales* order (Horse.32) were also partially reconstructed (Supplementary Table S1). An overview of taxa recovered from different datasets is in Figure 3.

### Annotation and Phylogenetic Relationship

In total, 35 hypothetical proteins were classified as members of IPR011118, IPR010126, IPR002921 entries (Table 2). None of the predicted proteins belonged to the IPR034429 entry. The BLASTP bitscores against reference FAEs were generally low, as were the sequence identities which ranged from 21 to 46% (Table 2). In the BLASTP search against the full non-redundant protein database, the scores were higher, with the sequence identities ranging from 27 to 100%. One of the predicted proteins (Horse.16: FOA763) identically matched to a bacterial FAE and two others (Horse.14: NAH160; Soil.2: DAH257) had slight similarities (32 and 50%, respectively) to bacterial FAEs. Five proteins of IPR002921 entry contained the serine active site motif and were thus considered as putative FAEs (Table 3). Taxonomic classifications of binned genomes with FAE coding capacities are in Table 4. Genomes of the *Clostridiales* order in the Cow and Horse datasets coded for FAEs, as did a genome of *Candidatus Rhabdochlamydia* genus in the Sediment dataset.

**TABLE 2:** BLASTP scores of putative proteins annotated as members of IPR011118, IPR010126, and IPR002921 entries of InterPro database.

			**BLASTP scores^a^**							
			**Reference FAEs**				**Non-redundant proteins of NCBI**			
**Sequence ID**	**Genome**	**InterPro signature**	**Matched hit**	**Bitscore**	**E.value**	**Identical match (%)**	**Matched hit**	**Bitscore**	**E.value**	**Identical match (%)**
**Cow dataset**										
ELP382	Cow.1	IPR002921	A2QSY5	31	3E-03	26	Hypothetical protein (WP_103986834.1)	245	2E-65	32
KFM882	Cow.4	IPR010126	A1CC33	32	8E-04	21	Hypothetical protein (WP_092995296.1)	861	0E + 00	97
GEF307	Cow.5	IPR010126	A1CC33	52	1E-10	27	Esterase (WP_122274278.1)	319	5E-106	55
JPJ405	Cow.7	IPR002921	A1CC33	24	3E-01	35	VWA domain-containing protein (WP_042172771.1)	184	3E-46	31
OJJ032	Cow.9	IPR002921	B8NIB8	30	6E-03	24	Hypothetical protein (WP_093044389.1)	453	2E-145	44
BPL864	Cow.11	IPR010126	Q0CDX2	49	2E-09	31	Hypothetical protein (CCX69434.1)	327	5E-105	40
**IDJ731**	Cow.15	IPR002921	Q0CBM7	32	1E-03	26	Hypothetical protein (WP_120429016.1)	223	4E-58	30
IDJ033	Cow.15	IPR002921	Q9P979	29	9E-03	30	Hypothetical protein (WP_081669054.1)	155	4E-37	34
EMM549	Cow.16	IPR010126	B8M9H9	38	7E-06	29	Hypothetical protein (WP_092995296.1)	488	6E-171	86
GMA315	Cow.28	IPR010126	G2QND5	53	7E-11	25	Hypothetical protein (WP_081861271.1)	457	2E-159	74
GMA314	Cow.28	IPR010126	A1CC33	48	2E-09	35	Hypothetical protein (WP_081861271.1)	433	5E-151	79
**Horse dataset**										
BEP156	Horse.7	IPR002921	Q0CBM7	39	3E-06	31	Lipase family protein (WP_087378587.1)	166	3E-44	31
**BEP310**	Horse.7	IPR002921	Q2UNW5	37	3E-05	22	Hypothetical protein (WP_073288296.1)	149	2E-33	36
BLI323	Horse.8	IPR010126	G2QND5	56	3E-11	31	Hypothetical protein (WP_028520965.1)	87	4E-17	65
NAH160	Horse.14	IPR011118	B8NPT0	103	1E-26	26	Tannase/FAE family α/β hydrolase (WP_106055381.1)	209	2E-58	32
FOA763	Horse.16	IPR010126	Q9Y871	223	2E-69	45	FAE (WP_101478763.1)	1023	0E + 00	100
FOA089	Horse.16	IPR010126	Q9Y871	245	1E-77	46	polyhydroxybutyrate depolymerase (RAR66513.1)	1036	0E + 00	100
FOA043	Horse.16	IPR010126	Q9Y871	81	4E-19	26	Carbohydrate-binding protein CenC (WP_101478973.1)	1009	0E + 00	100
IIC869	Horse.17	IPR010126	Q9Y871	27	2E-02	34	Hypothetical protein (WP_025834368.1)	322	1E-103	58
CCB829	Horse.19	IPR010126	Q9Y871	52	2E-10	26	Poly(3-hydroxybutyrate) depolymerase (CDA95053.1)	266	2E-85	46
OCA543	Horse.20	IPR010126	Q9HGR3	42	8E-07	25	Hypothetical protein (WP_117574921.1 or WP_118573219.1)	676	0E + 00	60
**LLA035**	Horse.22	IPR002921	Q0CBM7	25	1E-01	32	DUF2974 domain-containing protein (WP_073565233.1)	59	3E-06	36
**KKH736**	Horse.24	IPR002921	Q9P979	22	1E + 00	24	Lipase Class 3 (WP_014271472.1)	53	3E-04	27
KKH120	Horse.24	IPR002921	A2QSY5	32	2E-03	26	Lipase class 3 (WP_014271472.1)	108	4E-23	29
KKH742	Horse.24	IPR002921	Q0CBM7	31	1E-03	28	Hypothetical protein (WP_124756111.1)	136	2E-33	33
KKH437	Horse.24	IPR002921	Q0CBM7	25	2E-01	24	VWA domain-containing protein (WP_042172771.1)	210	6E-53	28
HEH134	Horse.25	IPR002921	B8NIB8	34	7E-05	29	Hypothetical protein (PWM34645.1)	163	3E-44	43
MMI830	Horse.30	IPR010126	G2QND5	63	9E-14	26	Phospholipase/carboxylesterase (EGG54990.1)	285	1E-83	42
BKD217	Horse.31	IPR010126	Q9HGR3	41	1E-06	27	Hypothetical protein (WP_093122987.1)	200	5E-58	39
JJL430	Horse.32	IPR002921	Q0CVS2	26	1E-01	39	Lipase Class 3 (CDC29637.1)	127	4E-27	36
GNF552	Horse.43	IPR010126	G2QND5	46	1E-08	28	Poly(3-hydroxybutyrate) depolymerase (CDD18994.1)	341	1E-113	58
**Sediment dataset**										
NEB278	Sediment.2	IPR002921	Q0CBM7	52	2E-10	31	Lipase family protein (RPJ12008.1)	656	0E + 00	95
**ELA265**	Sediment.5	IPR002921	B8NIB8	26	8E-02	24	Hypothetical protein (PWU16597.1)	950	0E + 00	78
**Soil dataset**										
DAH257	Soil.2	IPR011118	B8NPT0	179	9E-53	30	Tannase/FAE family α/β hydrolase (RZM34741.1)	491	5E-166	50
DAH259	Soil.2	IPR010126	G2QND5	33	3E-04	33	Hypothetical protein (OLB12881.1)	362	5E-121	59

*Only the highest scores are shown. Proteins annotated as putative feruloyl esterase (FAE), based on the possession of serine active site (PS00120), are in bold (see Supplementary Sequence File S1 for putative protein sequences). ^*a*^Bitscore, E.value, and identical match were respectively used to report subject sequences matched.*

**TABLE 3:** Location of the serine active site motif (PS00120) in putative feruloyl esterases (FAEs) (see Supplementary Sequence File S1 for complete sequences).

**Putative FAEs**	**Protein length^a^ (aa)**	**Motif location (aa)**	**Motif sequence**
IDJ731	613	162–171	VLLTGYSRGA
BEP310	684	197–206	IFITGHSRGA
LLA035	305	195–204	VYLTGHSLGG
KKH736	336	183–192	LYIIGHSLGS
ELA265	600	365–374	LEITGHSLGG

*^*a*^The length corresponds to the protein sequence without signal peptide, if any.*

**TABLE 4:** Taxonomic classification of binned genomes with feruloyl esterase (FAE) coding capacities.

**Recovered genomes**	**Putative FAEs**	**Bin size (MiB)**	**Completeness (%)**	**Contamination (%)**	**Assigned taxon**
Cow.15	IDJ731	1.7	69.56	3.52	o_Clostridiales
Horse.7	BEP310	1.7	90.88	0.79	g_Ruminococcus
Horse.22	LLA035	1.9	72.11	2.57	f_Lachnospiraceae
Horse.24	KKH736	1.1	69.13	0	f_Ruminococcaceae
Sediment.5	ELA265	0.8	49.81	1.19	g_Candidatus Rhabdochlamydia

**FIGURE 4:**
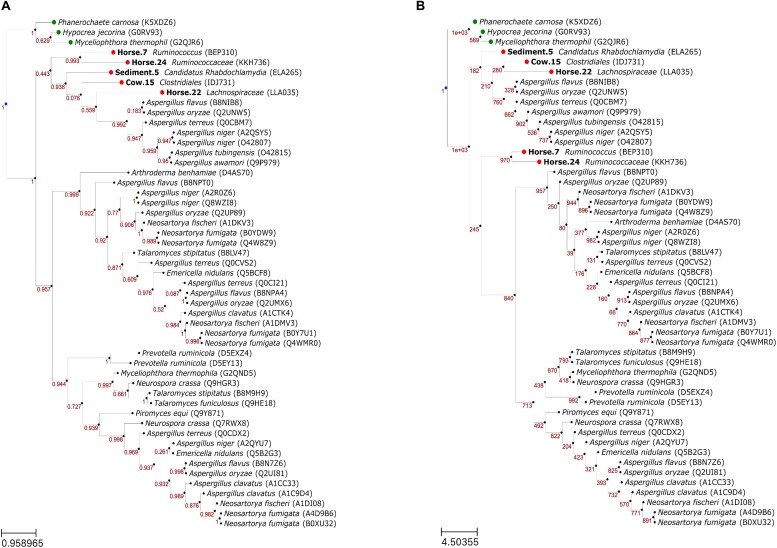
Phylogenetic relationships of feruloyl esterases (FAEs) predicted in this study (in bold) with reference FAEs, using maximum likelihood **(A)** and neighbor joining **(B)** methods. Confidence values in panels **(A)** and **(B)** represent Shimodaira–Hasegawa support values (1000 resampling) and bootstrap values (1000 bootstraps), respectively. Leaf nodes in green are the outgroup. Leaf naming is “<species> (<UniProt ID>)” for the reference/outgroup sequences and “<bin id><predicted taxon> (<protein id>)” for the putative sequences.

Overall, the topologies of ML (Figure 4A) and NJ (Figure 4B) trees were similar with formation of three main clades. In the ML tree, the putative FAEs and FAEs-A formed a clade that had a moderate support (0.443). The other two clades had high supports and collectively included FAEs-B, FAEs-C and a FAE-D (Q7RWX8) that was included in our reference dataset. The main difference in the NJ tree was that the putative FAEs of Horse.7 and Horse.24 were not placed with the other putative sequences and FAEs-A in one clade but were placed basal to the other two main clades.

## Discussion

In this study, we used whole-genome shotgun metagenomics combined with *de novo* assembly and genome binning to study prokaryotic FAEs of anaerobic (cow rumen, large intestine of horse and sediment of fresh water) and microaerobic (topsoil) ecosystems. The lower assembly quality in the Sediment and Soil datasets (Table 1) suggests that there was insufficient coverage of microbial genomes in these samples, likely due to a high microbial diversity in these two ecosystems, something evident from Figure 2.

Although several proteins from the binned genomes matched the FAE-containing entries of the InterPro database, these proteins showed very low primary sequence similarities to our reference FAEs. We therefore, explored the reference FAEs to identify sequence motifs of this enzyme family to enable a functional annotation of our predicted proteins. Surprisingly, only FAEs-A consistently contained a motif, i.e., the serine active site (PS00120), a signature of some lipases^4^. It was previously reported that FAEs-A have sequence similarities to lipases (Crepin et al., 2004) and therefore, this finding may not be entirely unexpected. The PS00120 motif is also detected in FAEs of *Lactobacillus* spp. (Xu et al., 2017). Our finding here indicates that attempts should be made to identify sequence features unique to FAEs to facilitate functional annotation of novel FAEs.

The PS00120 motif was only found in the protein sequences classified as members of IPR002921 domain but not in all of them (only 5 out of 16 sequences). As the IPR002921 entry describes a domain in FAEs-A, we considered these five sequences as putative FAE. The results from BLASTP search against non-redundant proteins (Table 2) suggest that these putative FAEs represent novel sequences in this enzyme family.

Species belonging to the *Clostridiales* order were previously reported to produce FAEs, including *Butyrivibrio fibrisolvens* (Dalrymple et al., 1996), *B. proteoclasticus* (Goldstone et al., 2010), *Ruminococcus albus*, and *R. flavefaciens* (McSweeney et al., 1998). To the best of our knowledge, this is the first report about FAE coding capacity within the *Candidatus Rhabdochlamydia* genus. It should be pointed out that although the Sediment dataset was constructed from a mix of different sources, the genome quality analysis showed that the considered genome had a very low contamination (∼1%, Table 4), indicating that our sampling strategy was adequate.

The association between specific activities of FAEs, summarized as the A–D classification scheme (Crepin et al., 2004), and phylogenetic relationships of these enzymes is not straightforward (Dilokpimol et al., 2016), something also evident from our phylogenetic analysis. In both ML and NJ trees, two clusters were formed with each comprising FAEs of mixed specific activities. It is possible that ecological niches and specific needs of individual species largely determine the specific activity of FAEs, also pointed out by Benoit et al. (2008). This was however not the case for the FAEs-A, as in both trees the FAEs-A formed a distinct cluster, not showing close evolutionary relationships with other types of FAEs. The different evolutionary lineage of FAEs-A is further evidenced from that the PS00120 motif was only found in this type of FAEs. Interestingly, three putative sequences were consistently clustered with FAEs-A in both phylogenetic trees, suggesting that these putative FAEs may have specific activities similar to this type of FAEs, something that should be verified experimentally. Production of FAEs-A is until now only found in fungi and in particular in *Aspergillus* spp.

## Conclusion

In total, 31, 44, 7, and 6 prokaryotic genomes were reconstructed from the Cow, Horse, Sediment, and Soil datasets, respectively, and were explored for FAE coding capacities. Four genomes of *Clostridiales* order in the Cow and Horse datasets and one genome of *Candidatus Rhabdochlamydia* genus in the Sediment dataset were found to have such capacity. In total, five FAEs were predicted. The results from BLASTP against non-redundant protein database of NCBI suggested that these putative FAEs are novel. Phylogenetic analysis suggested that at least three putative sequences might have specific activities similar to FAEs-A.

## Data Availability Statement

Raw data is deposited at the Sequence Read Archive database under PRJNA543979 accession number.

## Author Contributions

KM designed the study and did the sampling, DNA extraction, bioinformatic analyses and preparation of the first draft of the manuscript. JS provided bioinformatic expertise and contributed to the manuscript preparation.

## Conflict of Interest

The authors declare that the research was conducted in the absence of any commercial or financial relationships that could be construed as a potential conflict of interest.

## Footnotes

1 https://github.com/ParBLiSS/FastANI

2 https://prosite.expasy.org/PS00120

3 http://www.atgc-montpellier.fr/

4 https://prosite.expasy.org/PDOC00110

## Supplementary Material

TABLE S1 Genome quality and taxonomic classifications of recovered genomes searched for feruloyl esterase coding capacities.

SEQUENCE FILE S1 Putative protein sequences annotated as members of IPR011118, IPR010126, and IPR002921 entries of InterPro database. Sequences annotated as putative feruloyl esterases are IDJ731 (1–30), BEP310 (1–25), LLA035, KKH736 (1–22), and ELA265, with location of predicted signal peptides, if any, in parentheses.
